# Dielectric Dispersion Modulated Sensing of Yeast Suspension Electroporation

**DOI:** 10.3390/s22051811

**Published:** 2022-02-25

**Authors:** Guilherme B. Pintarelli, Jessica R. da Silva, Wuqiang Yang, Daniela O. H. Suzuki

**Affiliations:** 1 Department of Electrical and Electronics Engineering, Institute of Biomedical Engineering, Federal University of Santa Catarina (UFSC), Florianopolis 88040-370, Brazil; jessica.rodrigues@posgrad.ufsc.br (J.R.d.S.); daniela@ppgeel.ufsc.br (D.O.H.S.); 2 Department of Electrical and Electronic Engineering, The University of Manchester, Manchester M13 9PL, UK; wuqiang.yang@manchester.ac.uk

**Keywords:** pulsed electric field, bio-impedance, biological system modeling, bio-technology, bio-membranes

## Abstract

A specific pulsed electric field protocol can be used to induce electroporation. This is used in the food industry for yeast pasteurization, in laboratories for generic transfer and the medical field for cancer treatment. The sensing of electroporation can be done with simple ‘instantaneous’ voltage-current analysis. However, there are some intrinsic low-frequency phenomena superposing the electroporation current, such as electrode polarization. The biological media are non-homogeneous, giving them specific characterization in the broad frequency spectrum. For example, the cell barrier, i.e., cell membrane, causes so called β-dispersion in the frequency range of tens to thousands of kHz. Electroporation is a dynamic phenomenon characterized by altering the cell membrane permeability. In this work, we show that the impedance measurement at certain frequencies could be used to detect the occurrence of electroporation, i.e., dielectric dispersion modulated sensing. This approach may be used for the design and implementation of electroporation systems. Yeast suspension electroporation is simulated to show changes in the frequency spectrum. Moreover, the alteration depends on characteristics of the system. Three types of external buffers and their characteristics are evaluated.

## 1. Introduction

Pulsed Electric Field (PEF) is a non-thermal electricity-based process to control cells. PEF is used in bio-technology [1], veterinary [2] and medical research [3], and food industry [4,5]. These applications require specific levels of electroporation control. Techniques and sensing electroporation improve safety and optimization applications of PEFs. Recent progress on electroporation methods propose studies of PEF using nanoparticles [6], gels [7,8], microdevices [9], and electrospun PCL [10].

The PEF approach makes use of a combination of pulse amplitudes, pulse durations and pulse repetitions. Usually, high amplitude, short duration, and rectangular PEF (hundreds of kV/m and tens to hundreds of µs) is used for the permeabilization of the cell membrane, also known as electroporation or electro-permeabilization [11,12]. Electroporation can arise as reversible or irreversible, mainly depending on the PEF amplitude. If PEF is configured as low amplitude and long duration (tens of kV/m and tens ms), it is possible to primarily cause electrolysis, which may induce chemical ablation [13,14].

The electroporation theory is based on formation of nano-pores in the cell membrane due to excessive accumulation of transmembrane ionic charges. Pore hypothesis evidence is given by measurements showing the increase in ionic permeability due to PEF. Pore creation and expansion is described to change the membrane electrical properties (e.g., electrical conductivity) [15]. The pore number can be described by the Smoluchowski equation [16]. The membrane changes affect the macroscopic scale electrical properties, which can be modeled using a macroscopic current. This concept is mostly used to describe PEF tissue response, which enables the pre-treatment of PEF-based cancer therapies [17,18].

PEF is usually delivered in the signature of 100 µs rectangular pulses burst, which is described by diverse sub-4.4 kHz frequency components. Current models use instantaneous current-voltage measurements to describe the PEF electrical current [18,19], which is a sum of conduction current, dispersive effects, electrode-biological-media polarization, and electroporation. From the engineering point of view, differentiating the electroporation current from other PEF-current is complex. We call the electroporation current the superimposed non-physiological displacement current in the cell membrane due to pore opening. It is known that the biological systems are electrolytes and charges are subject to changes in polarization, i.e., orientation and displacement of charges. The polarization delay is given in the time domain, and it is called relaxation, which can be transposed to the frequency domain, so-called dispersion. Biological systems are characterized by the three most expressive relaxation factors: ions diffusion outside the cells, cell’s membrane interface charging, and molecule orientation, which contribute to Schwan’s dispersions, α, β, and γ, respectively [20]. The α-dispersion and electrode-biological-media-dispersion are located at sub-10 kHz, which is where the PEF burst energy spectrum is. Those phenomenon dynamics have not been completely understood, interfering in the direct current measurement.

Besides the complexity of characterizing biological samples using sub-10 kHz measurements, the electroporation itself is not entirely understood. Thus, PEF systems usually do not have electroporation feedback because there is no adequate PEF probing method. We have recently shown that it is possible to eliminate yeast using irreversible electroporation and the macroscopic current changes due to cell breakage [4]. Yeasts are a problem in the industry, as their contamination can cause financial loss. If contaminated yeast is injected into a human body, it can cause health problems. However, there are no efficient methods to sense electroporation for probing the actual electroporation current, which can be ultimately used to directly probe the membrane conductivity or electroporation pore density. In this work, we evaluated the effects of electroporation on the dielectric spectrum of cell suspensions. This evaluation can provide insights into the development of electroporation/PEF high frequency (>10 kHz) sensors. New sensors can be designed to operate in a frequency range adequate for detecting membrane changes while avoiding sub-10 kHz PEF direct current polarization disturbances. This technique provides more accurate readings of electroporation mechanisms to control and optimize industrial processes.

To demonstrate the feasibility of supervising electroporation, we propose a numerical study on the sensing of the suspension dielectric dispersion. Yeast cell suspension is known to have well-defined β-dielectric dispersion in the frequency range of 0.1–1 MHz due to the cell membrane. The electroporation model is used to calculate changes in the membrane conductivity (*σ_m_*) during electroporation. System invariance is assumed on a small scale, and the suspension dielectric spectrum is calculated. A computer model is used to demonstrate that the system dielectric dispersion changes due to the cells’ membrane and their interaction with the extracellular media and electroporation. This technique may be applied at a microsensor embedded at an industrial electroporation line or an electroporation lab on a chip device.

## 2. Materials and Methods

Analytical approaches to solving electric fields at the cellular level are based on spatially dependent, partial differential equations [21,22]. It is challenging to assume modeling conditions, such as irregular cell shape, nearby cells, and non-linear cell membrane change due to electroporation [23]. This research uses a finite element method (FEM) to deal with those conditions at a computational cost.

**Figure 1:**
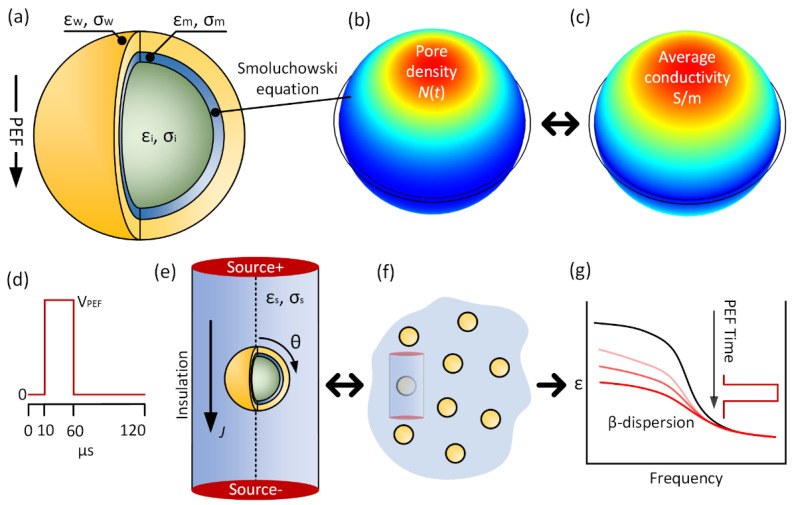
(**a**) Two-shell whole yeast cell model under PEF. PEF induces higher transmembrane voltages, which increases (**b**) pore density *N*(*t*) and (**c**) the average membrane conductivity. The color map values are as follows: red means higher and blue means lower. (**d**) The 50 µs stimulus signal profile, where V_PEF_ is calculated to obtain 400 kV/m maximum PEF. (**e**) An infinitesimal cylindrical model with one ‘electroporated’ cell. The θ angle is used to address membrane conductivity and transmembrane voltage results. (**f**) The infinitesimal cylinder describes a cell suspension with 1% yeast concentration ratio. (**g**) The decrease in the membrane conductivity affects the β-dispersion of the yeast suspension.

In the following, the effects of electroporation and extracellular buffers on the equivalent (bulk) electrical properties are evaluated theoretically. A two-shell model is used to represent the yeast cell (see Figure 1a) under 50 µs (10 to 60 µs) and 1 µs (2 to 3 µs), 400 kV/m PEF (see Figure 1d). The PEF induce higher transmembrane voltages at cell poles, which provokes electroporation. The electroporation model describes the pore density *N*(*t*) and average membrane conductivity due to PEF (see Figure 1b,c). The ‘electroporated’ yeast cell is in the center of an infinitesimal cylindrical volume (see Figure 1e). The cylinder is used to evaluate conduction current density *J* during PEF. The infinitesimal volume describes the yeast suspension (see Figure 1f). The data are calculated and analyzed in terms of the cell’s membrane conductivity, the transmembrane voltage, and equivalent relative permittivity *ε_eq_* of the infinitesimal cylinder during the PEF protocol. Membrane conductivity and transmembrane voltage are given as function of θ angle (see Figure 1e). The low-frequency current increases due to the cell’s membrane interface change, altering the sample’s electrical characteristic, i.e., β-dispersion.

### 2.1. Numerical Modeling

The time-harmonic conduction and displacement currents are solved according to Maxwell-Ampère’s law, as given by Equation (1). In this equation, it is assumed that all variations in time occur as steady-state sinusoidal signals. Because of a non-linear condition due to electroporation, the observation is considered to be time-dependent, and linearity is assumed over a small-scale step. Thus, the time step is a stationary problem in the frequency domain using complex values. The fields are represented by amplitude and phase (i.e., phasors), while the frequency is specified as a predefined sweep sinusoidal signal input. A frequency range from 100 to 1 MHz is employed, as they are the optimal frequencies for cell membrane sensing [24]:

(1) J=σE+jωεE

where *J* is the conduction current density and *E* is the electric field (V/m), computed as *E* = −∇*V* (where *V* is the electric potential) [25].

The whole cell geometry model is a two-shell yeast model located at a cylindrical spatial media. The model is 2D axisymmetric, and a revolution in the cylinder axis is used to obtain volumetric results (see the dotted line in Figure 1e). The volumetric model containing one cell is shown in Figure 1e. The whole cell simulation parameters and their description are given in Table 1. Cells of the yeast *Saccharomyces cerevisiae* are used because detailed electrical properties literature and their ellipsoidal shape are often approximated as a two-shell sphere [26,27,28]. In this research, a 1% yeast concentration ratio (i.e., the ratio between cell’s volume divided by the total solution) is used, which is approximately 2 × 10^8^ cells/mL. This is a typical yeast concentration from the experimental point of view [29]. The cell concentration is controlled by adjusting the cylinder volume, i.e., changing the total buffer volume to match the desired concentration.

COMSOL Multiphysics^®^ version 4.4 software is used for FEM modeling and 2D electric currents application (‘ec’, from the COMSOL’s AC/DC Module). The electroporation model is solved in the time domain (as described previously [4,31]), giving results of pore density *N*(*t*) and average membrane electrical conductivity (see Appendix A Equations (A1) and (A2)). The simulation steps are defined as linear and were 0.5 µs when using 50 µs PEF and 0.01 µs when using 1 µs PEF. The simulation is run until 120 µs (for 50 µs PEF) and 20 µs (for 1 µs PEF) to calculate post-PEF transient effect. The small signal frequency domain is used to calculate equivalent complex permittivity. The boundary conditions were insulation on the curved cylinder surface, and the top and bottom bases were used as sinusoidal and PEF voltage sources. There is a differential bias of 1 × 10^−4^ V between the top and bottom boundaries used for small signal analysis. We suppose that our simulation volume behaves as an infinitesimal volume in a whole yeast suspension as the gradient of the voltage in the insulation is less than 2.5% of the source voltage, and the gradient of the current in the top and bottom sources is less than 2.5%. Those parameters are critical and must be as small as possible. If the gradient in the boundary layers is negligible, then there is no field diffraction in the boundary so that we can assume an infinitesimal volume design model.

A custom mesh is designed for this study. The mesh is edge mapped for bilateral symmetry and mapped as 10 per 1000 quadratic elements for each cell’s membrane and wall. Other elements are made using COMSOL’s free triangular semiconductor ‘fine’ pre-set. The maximum element size is set as 3.3 × 10^−7^ m. The mesh consists of 171,416 tetrahedral elements. The transmembrane voltage due to PEF can be analytically calculated using the Laplace equation [32]. We validated the numerical calculations by comparing them to the analytical solution proposed by Gimsa and Wachner [33]. The maximum relative error between the analytical solution and the computer simulation was 5.18% (using the parameters shown in Table 1). Therefore, we considered our model to be sufficiently accurate for our study.

### 2.2. Yeast Cell Passive Properties

The electrical parameters of the tested materials are listed in Table 2. Low conductivity media (also called ‘electroporation buffer’) are preferred for mitigating electrolysis during PEF [34], and some buffer composition is reported to improve electroporation efficiency [35]. For the in vitro experiment, the conductivity and osmolality of the buffer can be modulated by changing salts (e.g., KCl and NaCl) and sugars (e.g., sucrose). However, caution must be taken for modeling, as the buffer conductivity influences the cell’s wall and membrane conductivity and intracellular conductivity [26,27,28]. We computed results using three conductivities situations: 1 × 10^−3^ S/m (low *σ*), 50 × 10^−3^ S/m (medium *σ*) and 0.1 S/m (high *σ*) buffer. It is found that the conductivity of laboratory deionized water is typically 1 × 10^−3^ S/m and final yeast’s suspension solutions in a range from 50 × 10^−3^ to 0.2 S/m [26]. The membrane channels state is ‘closed’ (i.e., the lower conductivity end at approximately 0.25 × 10^−6^ S/m) at 1 to 10 × 10^−3^ S/m buffers and ‘increasingly opening’ for buffers over 20 × 10^−3^ S/m. For a typical 50 × 10^−3^ S/m buffer [35], the membrane conductivity is approximately 0.1 × 10^−3^ S/m [27]. The cell wall is a known negative charged sieve-like structure. Thus, it is the first selective barrier [36]. Some reports say that for a highly conductive medium, the conductivity of the cell’s walls is approximately 10 to 20% of medium conductivity, which may be explained by the wall’s porosity and its charges [26]. The cytoplasm is known to be a highly conductive medium, as it holds the necessary salt and protein for a cell’s life.

## 3. Results

**Figure 2:**
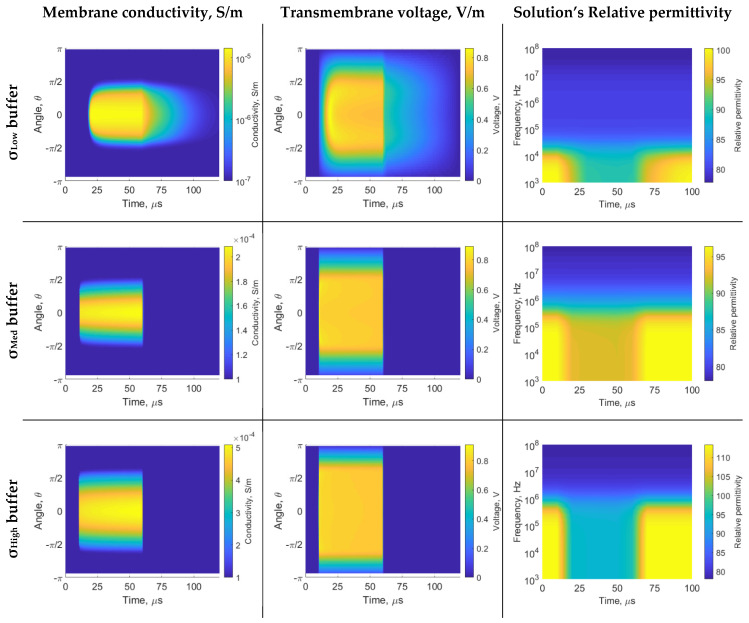
Results of 50 µs PEF: Membrane conductivity, transmembrane voltage and solution equivalent relative permittivity. The horizontal axis is the time in µs. The PEF starts at 10 µs and ends at 60 µs. The vertical axis of the membrane conductivity and transmembrane voltage figures represent the angle location in the membrane. The vertical axis of the solution relative permittivity figures is the sensing frequency (1 kHz to 100 MHz range).

The membrane conductivity, transmembrane voltage, and solution’s relative permittivity when using 50 µs PEF is shown in Figure 2. During 10 to 60 µs, the PEF is enabled. All buffers are adequate to induce electroporation transmembrane voltages. The *σ*_Low_ buffer has the slowest time dynamics (see the post-60 µs dynamism of membrane conductivity and transmembrane voltage). This occurs due to the poor buffer-cell interface conductive coupling, which leads to slower capacitive charging and discharging processes. The membrane state change is practically instantaneous when using *σ*_Med_ and *σ*_High_ buffers. Additionally, the extent of membrane area affected by electroporation is higher when *σ*_High_ buffer is used (approximately –π/2 to π/2).

**Figure 3:**
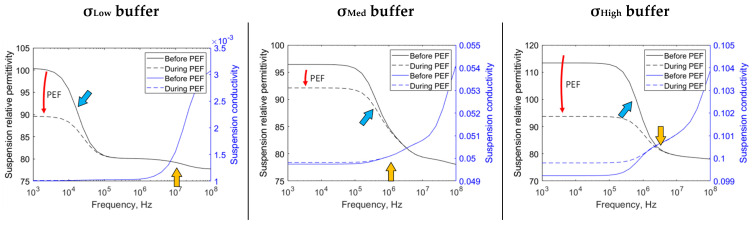
Cell suspension dielectric properties before and during PEF. The electroporation decreases the equivalent solution permittivity. The red arrow indicates the time direction. The blue arrow indicates the membrane main-β-dispersion. The gold arrow indicates the yeast wall peak sub-β-dispersion.

Figure 3 shows the cell suspension dielectric properties before and during electroporation, using data from 8 µs and 50 µs time step, respectively (from 50 µs PEF). The membrane interface changes due to electroporation and affects the β-dispersion magnitude. The dispersion center depends on the system’s characteristics; the dispersion center is approximately 10 kHz for *σ*_Low_ buffer and 500 kHz for *σ*_Med_ and *σ*_High_ buffer (see the blue arrows at Figure 3 solution’s relative permittivity). Frequencies higher than 1 MHz are not suitable for sensing the membrane dispersion, as the displacement current can flow through the cell, which is called ‘electrically invisible’. The β-dispersion is mainly explained by the cell barrier (membrane and wall). The cell wall is described to be electrically distinct from the membrane. Thus, a sub-β-dispersion is provoked by the cell wall and interfere with the ‘main’ β-dispersion. The wall interference starts at 0 Hz when using *σ*_Med_ and *σ*_High_ buffer, as the wall is less conductive than external media. The wall primarily impacts the *σ*_Med_ buffer β-dispersion as electrical characteristics of wall and membrane are more similar than *σ*_High_ buffer. The wall sub-β-dispersion has its peak indicated by the gold arrows in Figure 3 (gold arrows show the peak trans-wall voltage).

**Figure 4:**
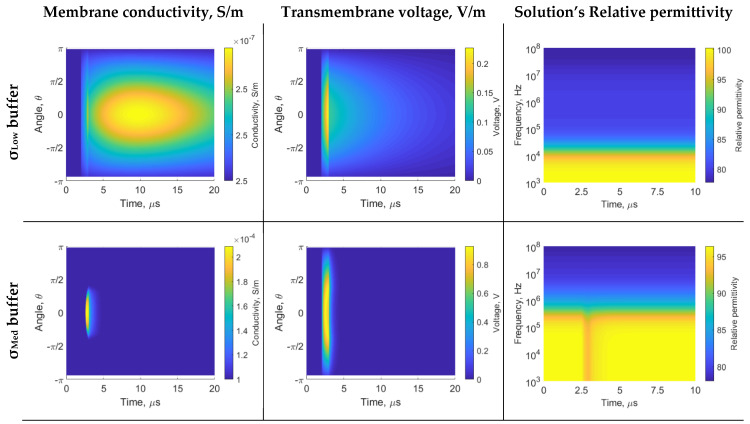

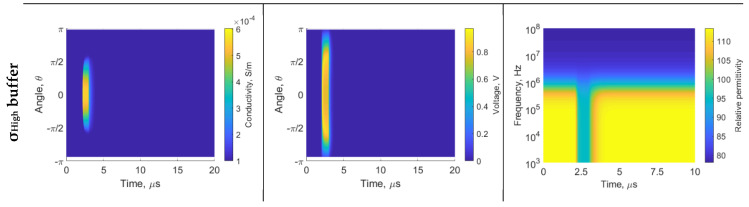
Results of 1 µs PEF: membrane conductivity, transmembrane voltage and solution equivalent relative permittivity. The axis description is used within Figure 3. The PEF starts at 2 µs, and ends at 3 µs. When using *σ*_Low_, the membrane conductivity changes are lower than 0.1%.

The 1 µs PEF results are shown in Figure 4. During 2 to 3 µs, the PEF is enabled. The *σ*_Low_ buffer slow dynamic cannot charge the membrane sufficiently for electroporation (the membrane charges up to 200 mV). Thus, the membrane conductivity change is negligible, and the spectrum does not change significantly (i.e., unsuccessful electroporation). The *σ*_Med_ buffer can charge the membrane sufficiently for electroporation. However, the 1 µs does not saturate conductivity, as seen in 50 µs PEF (Figure 2). Thus, *σ*_Med_ buffer β-dispersion decays only at the end of the 1 us pulse.

## 4. Discussion

This study is focused on the impact of cells’ relative parameters on the frequency spectrum of impedance, particularly how individual electrical properties affect the dielectric β-dispersion center. The β-dispersion is mostly described by the cell membrane interface. The dielectric dispersion dynamics can be used as a design parameter for micro/nano dielectric dispersion modulated sensors. Those sensors may be used for industrial irreversible electroporation or lab on a chip electroporation device [9]. FEM simulation has been used to find the equivalent *ε*, which depends on the inhomogeneous media. We have shown that an impedance sensor operating up to 1 MHz can perceive the alteration caused by electroporation in cells if the buffer conductivity is higher than 50 mS/m. An infinitesimal volume has been used to simulate a cell suspension, which is a reasonable approach for low density suspension. The electroporation analysis of cells attached or very closed to the microelectrodes [9] can use similar dielectric dispersion dynamics modulated sensors. However, specific cell geometry and properties, cell density, and electrode/chamber design may be simulated to determine appropriate sensor operating frequency and minimum buffer conductivity. The electroporation model is used to assess the membrane conductivity during PEF. The change in membrane conductivity affects the overall, i.e., ‘equivalent’, properties.

The maximum membrane conductivity for *σ*_Low_, *σ*_Med_, and *σ*_High_ buffers are 0.01 mS/m, 0.2 mS/m, and 0.5 mS/m, respectively, as shown in Figure 2. Even though we did not replicate the recent electroporated membrane conductivity of 3 mS/m to 30 mS/m [37,38,39], our results are consistent with some early experimental and theoretical works (0.01 mS/m to 1 mS/m) [32,40,41,42]. Ramos et al. [43] shows a membrane conductivity of 1 μS/m to 0.01 mS/m (conversion $\sigma_{m}=h\cdot G_{m}$ [44]) for yeast cells with 0.28 volumetric fraction. These reduction of electroporated membrane conductivity may be caused by volumetric fraction [45]. The present studies are similar to very low cell density, without reduced transmembrane potential caused by cells proximity. Despite the limitations, our findings are consistent with the previous reports of membrane conductivity.

The *σ*_Low_ buffer, i.e., deionized water, is a ‘reasonable’ insulator from a biological media perspective. We have observed that some cell types may not be compatible with this medium’s osmolarity. The electrical characteristics of this buffer are similar to the cell wall. Therefore, the effects of the buffer-wall interface are reduced, i.e., diffraction of the electric field and wall voltage drop. Because most of the dispersion is created only by the cell membrane, it tends to have the dispersion center at low frequency due to the thin membrane (the dispersion center, in this case, is near 10 kHz). The *σ*_High_ medium is more typical for liquid foods [4,38,46]. The *σ*_Med_ and *σ*_High_ buffers are more similar regarding dispersion center location. Those have the dispersion center near 500 kHz. From the membrane sensing point of view, it is interesting to see that the cell’s interface dispersion is higher than 10 kHz, as frequencies sub-10 kHz are probably contaminated with the chambers’ electrolytic double layer effect, i.e., electrode polarization and α-dispersion [26]. Therefore, this sensing approach can mitigate the unwanted impact by using measurements higher than 100 kHz. However, we cannot exceed 1 MHz, as the membrane and wall become ‘electrically invisible’. In other words, the voltage drop due to the cell’s interface is primarily linear. We did not notice changes in the spectrum at the time of electroporation (see the relative permittivity in Figure 2). Other researchers reported similar values [23,39,47].

The electroporation is modeled as the change in membrane conductivity due to the transmembrane voltage. When the transmembrane voltage reaches a certain value (~0.2–1 V) [48], membrane permeability increases as a consequence of the formation of hydrophilic pores in the lipid bilayer, leading to an increase in membrane conductivity (see Appendix A Equation (A2)). The *σ*_Low_ buffer has slower membrane (dis)charging dynamics (see the transmembrane voltage dynamics in Figure 2). Therefore, there is a direct effect on the conductivity of the membrane and the overall relative permittivity. For applications of molecule transfer, using a low conductivity buffer may be interesting to provide a slower dynamic transfer window, which is easier to operate from a control system perspective. Some authors have shown that a low conductivity buffer affects electroporation or enhances cell uptake [49,50,51]. Furthermore, the low conductivity buffer is attractive, as it results in lower electrical currents, and consequently, mitigates thermal or electrochemical effects. For purely irreversible electroporation PEF or arc plasm applications, this is the most suitable buffer to destroy the cell’s membrane. We observe that caution is needed if using fast PEF protocols. The 1 µs was not adequate to provoke electroporation when using *σ*_Low_ buffer. Additionally, the 1 µs PEF combined with *σ*_Med_ buffer is not fast enough to saturate the transmembrane voltage; thus, it may be the approximate lower threshold conductivity buffer to be used with 1 µs PEF. The dielectric dispersion sensing mechanism is capable of detecting the electroporation state: there are no alterations at *σ*_Low_ buffer (unsuccessful electroporation), late alterations at *σ*_Med_ buffer (caution state), and alterations at *σ*_High_ buffer (successful electroporation, see Figure 4).

The two-shell models are widely used in scientific research. However, they rely on various assumptions, e.g., spheric cells, nothing embedded in the wall or membrane, and homogeneous intracellular media [30]. Those may be a limiting factor in the analysis above 10 MHz [26,28]. However, this work, as well as others in the literature, have demonstrated that to sense the cell’s primally barrier (i.e., cell membrane), we should not exceed approximately 1 MHz [23,24,39]. The sub-β-dispersion (see Figure 2 gold arrow) is caused by the cell wall. The yeast experimental and simulations results described by Asami attributes sub-dispersions due to cell wall and vacuole [26]. It is known that the entire spectrum is interdependent (as dispersive effects overlap) [52], and PEF is reported to induce permeabilization in the entire cell barrier [53]. Therefore, we believe that at present, it is appropriate to evaluate the electroporation as a sum of effects (mixed results of membrane and wall). Even if the information generates a discrete event, it would be suitable in some scenarios, such as cold pasteurization by PEF. The membrane electroporation state change is almost instantaneous with a medium or high conductivity buffer. Thus, if a single pulse is used in these cases, the reversible electroporation may be modeled as a discrete event.

## 5. Conclusions

Yeasts may contaminate liquid food, causing financial or health problems. The PEF systems can eliminate microorganisms. However, those systems lack a directly electroporation feedback. This type of sensing is complex, as other phenomena result from direct current in an ionic media, i.e., electrode polarization. Disturbances in the spectrum due to electrode polarization are usually up to 10 kHz. We have demonstrated that it is feasible to supervise electroporation using impedance. The biological media are characterized by the β-dispersion, which is provoked mainly by the cell membrane. If the cell membrane changes due to electroporation, the biological media dispersion changes. A numerical study of a yeast suspension process can calculate the dielectric dispersion due to the cell barrier, external medial (buffer), and electroporation. Yeast cell suspension β-dispersion reduces due to the increase in the membrane conductivity. This approach may be used to develop a microsensor for use on an industrial electroporation line or an electroporation lab on a chip device.

## Figures and Tables

**Table 1:** Geometric simulation parameters.

Symbol	Value
Infinitesimal volume cylinder heigh	30 µm
Infinitesimal volume cylinder radius	16.8 µm
Cell wall thickness	220 ηm ^1^
Cell membrane thickness	8 ηm ^1^
Cell radius	4 µm ^1^

^1^ Data from [30].

**Table 2:** Non-linear electroporation model, electrical and geometric parameters of the simulation.

Parameter	*σ*_Low_ Buffer	*σ*_Med_ Buffer	*σ*_High_ Buffer
External solution conductivity (*σ_s_*)	1 × 10^−3^ [S/m]	50 × 10^−3^ [S/m]	0.1 [S/m]
External solution permittivity (*ɛ_s_*)	78	78 ^1^	77
Cytoplasm’s conductivity (*σ_i_*)	0.2 [S/m]	0.55 [S/m]	0.6 [S/m]
Cytoplasm’s relative permittivity (*ɛ_i_*)	50	50 ^1^	58
Initial membrane’s conductivity (*σ_m_*_0_)	0.25 × 10^−6^ [S/m]	0.1 × 10^−3^ [S/m]	0.1 × 10^−3^ [S/m] ^2^
Membrane’s relative permittivity (*ɛ_m_*)	6	7.6	5.2
Cell wall’s conductivity (*σ_w_*)	14 × 10^−3^ [S/m]	5 × 10^−3^ [S/m]	20 × 10^−3^ [S/m]
Cell wall’s relative permittivity (*ɛ_w_*)	60	60 ^1^	60

^1^ Data were not informed in [27]. We used data from [30]. ^2^ Data were considered as 0 S/m in [26]. We considered 0.1 × 10^−3^ S/m, which is the minimum physiological conductivity for yeast membranes.

## Appendix A

A non-linear model of electroporation can be used to simplify the Smoluchowski equation to describe the pore density *N*(*t*), as [54]:

(A1) $$\frac{dN\left(t\right)}{dt}=\alpha e^{{\left(\frac{V_{m}\left(t\right)}{V_{\mathrm{ep}}}\right)}^{2}}\;\left(1-\frac{N\left(t\right)}{N_{0}}e^{-q{\left(\frac{V_{m}\left(t\right)}{V_{\mathrm{ep}}}\right)}^{2}}\right)$$

where *V_m_*(*t*) is the transmembrane voltage, *V_ep_* is the electroporation voltage, *N*_0_ is the pore density at *V_m_* = 0 V, and α and q are constants. Equation (A1) was implemented in COMSOL using the Weak Form Boundary PDE physics application mode on all membrane surfaces.

The regions where pores are formed present average conductivity of [42,45,55]:

(A2) $$\sigma_{m}\left(t\right)=\sigma_{m_{0}}+N\left(t\right)\sigma_{p}\pi r_{p}^{2}K$$

where *σ_m_*_0_ is the membrane conductivity without electroporation, *σ_p_* is the conductivity of pore, *r_p_* is the pore radius, and *K* is:

(A3) $$K=\frac{e^{\upsilon_{m}}-1}{\frac{w_{0}e^{w_{0}-\eta \upsilon_{m}}}{w_{0}-\eta \upsilon_{m}}e^{\upsilon_{m}}-\frac{w_{0}e^{w_{0}+\eta \upsilon_{m}}+\eta \upsilon_{m}}{w_{0}+\eta \upsilon_{m}}}$$

where *w*_0_ is the energy barrier inside the pore, *η* is the relative entrance length of the pore, and $\upsilon_{m}=\frac{q_{e}}{kT}V_{m}$ is the non-dimensional transmembrane voltage.

The pore conductivity is [56]:

(A4) $$\sigma_{p}=\frac{\sigma_{w}-\sigma_{i}\;}{\ln\left(\frac{\sigma_{w}}{\sigma_{i}}\right)}$$

The cell and electroporation model parameters are given in Table A1.

**Table A1:** Non-linear electroporation model, electrical and geometric parameters of simulation.

Parameter	Symbol	Value	References
External conductivity	*σ_e_*	See Table 2	-
External permittivity	*ε_e_*	See Table 2	-
Membrane permittivity	*ε_m_*	See Table 2	-
Wall membrane conductivity	*σ_w_*	See Table 2	-
Wall membrane permittivity	*ε_w_*	See Table 2	-
Cytoplasm conductivity	*σ_i_*	See Table 2	-
Cytoplasm permittivity	*ε_i_*	See Table 2	-
Cell radius	*R*	5 μm	[30]
Thickness membrane	*h_m_*	8 nm	[30,57]
Thickness wall	*h_w_*	220 nm	[27,30]
Electroporation constant	*q*	2.46	[54,55]
Electroporation constant	*α*	109 m^2^s^−1^	[42,54,55]
Pore density at V_m_ = 0 V	*N*_0_	1.5 × 10^9^ m^−2^	[42,54,55]
Electroporation voltage	*V_ep_*	258 mV	[42,54,55]
Pore energy barrier	*W*_0_	2.65	[42,54,55]
Relative length of the pore	*η*	0.15 nm	[42,54,55]
Pore radius	*r_p_*	0.8 nm	[42,54,55]
Boltzmann constant	*k*	1.38 × 10^−23^ m^2^kgs^−2^K^−1^	[42,54,55]
Temperature	*T*	295 K	[42,54,55]
